# Supplementary material for: A multi-step nucleation process determines the kinetics of prion-like domain phase separation
Source: Nat Commun. 2021 Jul 23;12:4513. doi: 10.1038/s41467-021-24727-z (PMC8302766; doi:10.1038/s41467-021-24727-z)
Supplement: Supplementary file 1 — Supplementary Information [file 41467_2021_24727_MOESM1_ESM.pdf]

Supplementary Information

A multi-step nucleation process determines the kinetics of prion-like domain phase separation

Erik W. Martin[1]*, Tyler S. Harmon[2], Jesse B. Hopkins[3], Srinivas Chakravarthy[3], J. Jeremías Incicco[4,5], Peter Schuck[6], Andrea Soranno[4,5], Tanja Mittag[1]*

[1]Department of Structural Biology, St. Jude Children's Research Hospital, Memphis, TN
[2]The Max Planck Institute for the Physics of Complex Systems, Dresden, Germany
[3]The Biophysics Collaborative Access Team (BioCAT), Department of Biological Sciences, Illinois Institute of Technology, Chicago, IL
[4]Department of Biochemistry and Molecular Biophysics, Washington University in St. Louis, St. Louis, MO
[5]Center for Science and Engineering of Living Cells (CSELS), Washington University in St. Louis, St. Louis, MO
[6]Dynamics of Macromolecular Assembly Section, Laboratory of Cellular Imaging and Macromolecular Biophysics, National Institute of Biomedical Imaging and Bioengineering, National Institutes of Health, Bethesda, MD

* Corresponding authors: Erik W. Martin, erik.martin@stjude.org; Tanja Mittag, tanja.mittag@stjude.org

Supplementary Methods

Small angle x-ray scattering measurements

SAXS data were collected at the BioCAT (18ID-D) beamline at the Advanced Photon Source at Argonne National Laboratory[1]. The data were collected using a monochromatic x-ray beam with a wavelength of 1.033 Å (12 keV). The beam was microfocused using a 2D compound refractive lens to 20 x 5 um (H x V). All measurements were performed at room temperature (~22 °C). The data presented herein were collected over the course of two APS cycles with minor differences in hardware geometry. Chaotic-flow data for A1-LCD were collected with a camera length of 2067.0 mm and a nominal q-range of 0.007-0.6082 $Å^{-1}$. Chaotic-flow data on the aromatic depleted LCD and laminar-flow data were collected with a camera length of 2008.9 mm and a nominal q-range of 0.0096-0.6148 $Å^{-1}$. However, the usable q-range is limited by signal-to-noise and masking. The lowest analyzed angle was 0.01 $Å^{-1}$. Chaotic-flow data was collected during a 50 ms period with 45 ms exposures. Laminar flow data was collected with a 100 ms period with 95 ms exposures. The chaotic flow mixer pathlength is 0.25 mm and the laminar flow mixer pathlength is 1.025 mm.

The time resolved experiments yielded tens of thousands of individual images for each measurement. Masks were made for each position on the mixer to account for positional variations in scattering from the mixer itself. After masking, data were reduced to 1D profiles using a custom python script that called the radial averaging capabilities of BioXTAS RAW[2], version 1.6.0/1.6.3) using a beta version of the command line API for that program. Experimental uncertainty was initially calculated assuming a Poisson distribution of counts on a per-pixel basis for each image. Uncertainty was then propagated through radial averaging and all subsequent operations on the 1D profiles including averaging, subtraction and binning in q space, using standard addition in quadrature. All methods used were implemented in the BioXTAS RAW software and represent the standard treatment of uncertainty for SAXS data.

For both the chaotic-flow and the laminar-flow mixers, data were subtracted point by point, using identical physical positions on the mixer for buffer and sample profiles to account for positional variations in scattering from the mixer itself. For the laminar-flow mixer, measurements consisted of a 2D scan, both parallel to the direction of flow in discrete steps, yielding specific timepoints, and continuous scans perpendicular to the flow direction at each time point. The perpendicular scans at each time point were necessary as the position of the sample core stream in the channel can vary slightly due to small fluctuations in the flow rate of either buffer

or sample, so measuring a range of positions around the expected position ensures measurement of the sample. The position of the sample core stream in each perpendicular scan was automatically identified as the three contiguous positions with maximum intensity. The sample range was then manually verified to account for possible failure of the automated method due to outliers. After buffer subtraction, these three positions were averaged together to create a single scattering profile for a given time point.

Chaotic-flow TR-SAXS analysis

Chaotic-flow TR-SAXS data was collected using a microfluidic mixer similar to previous designs[3,4]. Data in chaotic-flow experiments was acquired as a function of position on the microfluidic mixer after complete mixing. A total of 152 frames were collected spanning times between 0.042 – 17.9 ms after mixing. Data was binned into 27 bins to increase the signal and each binned point was associated with the mean time after mixing within that bin. The binning rate was determined such that there was minimal evolution of the form factor over the binned time. Buffer subtraction was performed by recording data sets with identical parameters but with matched buffer not containing any protein injected into the mixer. Detector images were individually masked at each position along the mixer in order to eliminate any artifacts from local defects in the mixer. The final analyzed data was the average of two independent measurements. Data were recorded on different days for A1-LCD and the aromatic depleted LCD. The mixers used were different copies of the same design. Before each measurement, cytochrome c refolding kinetics were measured as a control (Supplementary Figure 2).

The R_G and I_0 values at each data point were calculated in two ways. First, the ATSAS program AUTORG[5] was used as a rough guideline to batch-process multiple data files. The default parameters for AUTORG were modified such that the Guinier fit only considered data where $qR_G < 1$. Final binned data were analyzed using a molecular form factor for unfolded proteins[6]. The form factor was empirically derived by Riback et al. by simulating poly-alanine peptides of varying length and Cβ attractive potential. The resulting form factor is an interpolation between dimensionless calculated SAXS profiles where the only fitting parameters are R_G and the Flory scaling exponent. Due to the poor signal-to-noise inherent in the time-resolved measurements, the default parameters for the form factor fit were modified such that only the angles $0.01\ \text{Å}^{-1} < q < 0.12\ \text{Å}^{-1}$ were used in the fit. The evolution of R_G^2 with time was modeled using an equation with the sum of single exponential decay and growth regimes:

$$R_G^2 = A_1\, e^{-\frac{t}{\tau 1}} + A_2\left(1 - e^{\frac{t}{\tau 2}}\right) + C_1 \qquad (1)$$

The growth regime was modeled as $1 - e^{\frac{t}{\tau 2}}$ because the data appeared to converge toward a metastable cluster size distribution prior to nucleation.

Supplementary Note 1: A1-LCD Solvation

The chaotic-flow mixing experiments revealed that as R_G decreased in the first milliseconds after mixing, I_0 paradoxically increased (Supplementary Figure 4). An increase in I_0 can be a sign of either oligomerization or increased concentration[7]. In light of the fact that the R_G decreases, oligomerization would imply that a monomer would have to collapse significantly and fold. However, we observed no evolution of the form factor (Supplementary Figure 3) and the protein remains unfolded. The fact that the protein flow is continuous means that all regions of the mixer should have identical concentrations. Mixing inhomogeneities were ruled out with the following experimental controls: Cytochrome c refolding experiments were used to confirm that the mixer was functioning properly (Supplementary Figure 2). Refolding times of cytochrome c are well established and were used to benchmark the performance[8]. Experiments with both copies of the chaotic flow mixer used in this study agreed with previously published refolding times. Further, the aromatic-depleted LCD is similar in size but lacks the ability to phase separate[9]. Data from this variant shows no change in either R_G or I_0.

Given that we have ruled out artifacts, we assume that the difference in I_0 must stem from a change in excess electron density associated with smaller conformations of A1-LCD. The chaotic-flow TR-SAXS experiments reported here are insufficient to determine the source of this excess density with certainty alone, but we propose that a change in solvation properties is consistent with the observed results. The hypothesis is that extended conformations of a disordered protein will have fewer stably bound solvent molecules than compact forms. As a disordered protein adopts more compact conformations, cavities with solvent-mediated semi-stable contacts will form resulting in a higher solvation density.

A model of a single sphere consisting of a core and a shell (a so-called core-shell sphere[10]) was used to explore whether R_g and I_0 can change in opposite directions if the shell density changes relative to the core density. The shell has a lower electron density due to the lower probability of amino acids occupying this space. The scattering intensity of such a core-shell sphere is given by:

$$I(q) = \frac{\phi}{V_s}\left(\frac{3}{V_s}\left[V_c\Delta\rho_{core-shell}\frac{\sin(qr_c)- qr_c\cos(qr_c)}{(qr_c)^3} + V_s\Delta\rho_{shell-solvent}\frac{\sin(qr_s)- qr_s\cos(qr_s)}{(qr_s)^3}\right]\right)^2 \quad (2)$$

In this model ϕ is the volume fraction, V_s is the sphere volume and r_s is the sphere radius, V_c is the core volume and r_c is the radius of the core. The total volume of the core-shell sphere is held constant and thus the simulated data neglects ϕ. Differences in mass are accounted for in the contrast term, ρ. The relative excess electron densities are given by $\Delta\rho_x$. The effect of increasing the excess electron density due to solvation in compact conformations can be approximated by varying the excess electron density of the core versus the shell ($\Delta\rho_{core-shell}$). Increasing the excess core density has the combined effect of decreasing the R_G due to a greater fraction of the mass being located near the center of mass and increasing the I_0 due to the overall greater density (Supplementary Figure 5a,b).

This problem can also be approached via ensemble modeling. We used a minimal ensemble (arbitrarily set to 6 conformations) to explore the effect of differences in solvation due to different radii. The conformations were chosen to represent the mean and the 25% most compact and most extended structures from the full simulated ensemble. Scattering profiles were calculated for each of 6 conformations using Crysol[11]. Two sets of scattering profiles were calculated with the 'dro' parameter, which represents scattering density of the solvation shell, set to the default value of 0.03 e/Å^3 for 'high contrast' or 0.015 e/Å^3 for 'low contrast'. The impact of modifying the solvation shell density by this amount is shown in Supplementary Figure 5c,d. In order to mimic the effect of a larger stable hydration shell for smaller conformations, scattering curves were combined such that the 4 conformations with the highest R_G values were assigned 'low contrast' and the 2 most compact conformations were assigned 'high contrast'. By effectively increasing the intensity of compact conformations, this combination resulted in a smaller average R_G and higher I_0 than when curves were calculated using a uniform low contrast and only modestly lower than curves calculated using high contrast for the entire ensemble (Supplementary Figure 3d).

The experiments presented in this work were not designed to probe solvation effects and they lack the information to precisely measure these effects, but we were motivated by the unexpected anticorrelation of R_G and I_0 to propose a possible physical explanation. While not conclusive, we have demonstrated that the experimentally observed results are in agreement

with a model in which compact unfolded protein conformations have a solvation shell that is relatively more stable than that of extended conformations of the same protein. It is important to note that this effect it due to an increase in excess contrast from the solvation shell. If an additional increase in contrast arises from ion adsorption or an enhanced ionic double layer, this would also be consistent with the models.

Supplementary Note 2: Assembly metric analysis of Laminar-flow TR-SAXS data

In laminar-flow TR-SAXS experiments, we used a previously described mixer and the data was also collected as a function of position after mixing (Figure 6)[12]. Unlike in chaotic-flow experiments where data was collected by moving the mixer perpendicular to the x-ray beam, in the laminar-flow experiments, the mixer was moved in a stepwise fashion and each measurement was a vertical scan of the channel. Data from the center of the protein stream was located in each measurement by finding the maximum integrated scattering intensity in the vertical scan. The final data for each measurement was the average of the three exposures with the highest intensity during the vertical scan. The flow rate in the protein channel is approximately 0.96 μL/sec or 186 mm/sec. The beam is focused to 20 μm in the horizontal direction and each exposure is 0.095 seconds. Therefore, the flow is fast relative to the exposure time and each data point captures the ensemble of molecules in approximately 3 x 0.096 μL.

Laminar-flow experiments at low NaCl concentration (200 mM), from which the R_G could be extracted, were analyzed using AUTORG[5] with the Guinier region optimized such that $qR_G < 1$. AUTORG provided the R_G and uncertainty for all datapoints. At higher NaCl concentrations, the protein assembles into larger species, whose contribution to the scattering intensity in the Guinier region makes it impossible to extract accurate R_G values. Instead, we sought to calculate an empirical metric that quantifies the degree of assembly.

The experimental data is analyzed by first fitting the earliest time points to a Gaussian coil form factor (as in Supplementary Equation 5 below). This is set as the 'monomer' form factor. The remaining frames are then aligned with the monomer form factor using robust linear regression at values of $q > 0.04$ Å^{-1}. The assembly metric is then calculated as the difference between the mean intensity of the experimental data and the calculated monomer form factor in the q range of 0.008 Å$^{-1} > q > 0.01$ Å^{-1}. Given the high scattering intensity at the smallest angles and that the assembly metric is the average of 39 data points, the values can be calculated with extreme

precision and the uncertainty is below the size of the markers in all datasets. There are other potential sources of uncertainty that are difficult to quantify, such as the quality of the normalization, so we chose to exclude mention of uncertainty in the figures. Any additional sources of uncertainty are likely small as well due to the high signal to noise in the region of the curves that is analyzed. Therefore, we conclude that the variation in the assembly metric between measurements at a given time point is resultant from real variations in the volume fraction of assembled protein and not simply a lack in precision in the data.

To test whether the assembly metric reflected the volume fraction of assembled protein, we tested its utility on synthetic data. The data in laminar-flow experiments at higher NaCl concentrations can be represented by the combination of a Gaussian chain form factor which accounts for the combination of monomer and small oligomers combined with a Lorentzian-like scattering function with a correlation length (ξ) longer than the resolution of the SAXS experiment (>50-100nm). Under this condition, the scattering at small angles decays simply as a power law with the exponent *d.*[13] The following function was used to fit the data with the Gaussian component scaled by A, the Lorentzian component scaled by B and parasitic, background, scattering accounted for with the constant C.

$$P(q) = A\,\frac{2\left[e^{-(qR_G)^2}-1+(qR_G)^2\right]}{(qR_G)^4} + B\,\frac{1}{1+(q\xi)^d} + C \tag{3}$$

In order to qualitatively model such a system, we considered a mixture of monodisperse Gaussian coils[10,14] and fuzzy colloids. The Gaussian coils were assigned a volume fraction of 0.001 which is on the order of the experimental conditions. We further assumed that the assemblies appearing early during phase separation would be similar to fuzzy colloids with a length scale larger than the resolution of the experiment. Therefore, we represent nucleation by converting a fraction of the volume of monomers into a volume of polydisperse fuzzy colloids. The size distribution is Gaussian and the final form factor is[15]:

$$I(q) = P_{GaussianCoil}(q) + P_{FuzzyColloid}(q) \tag{4}$$

$$P_{GaussianCoil}(q) = \frac{\phi_{monomer}M_w}{N_A\delta}\,\Delta\rho^2\,\frac{2\left[e^{-(qR_G)^2}-1+(qR_G)^2\right]}{(qR_G)^4} \tag{5}$$

$$P_{FuzzyColloid}(q) = \phi_{FC}V_c\Delta\rho^2\left(3\left[\frac{\sin(qr)- qr\cos(qr)}{(qr)^3}\right]e^{\frac{-(\beta q)^2}{2}}\right)^2 \tag{6}$$

$$P(r) = \frac{1}{\sqrt{2\pi\sigma^2\langle r\rangle^2}}\,e^{\frac{-(r-\langle r\rangle)^2}{2\sigma^2\langle r\rangle^2}} \tag{7}$$

For simplicity, the same electron density contrast ($\Delta\rho$) of 0.015 e/Å^3 was used for both Gaussian coils and colloids. The distribution of radii (r) is given by $P(r)$ with the mean value set to 1000 Å and the width (σ) as 0.1. The 'fuzziness' is defined by β which determines how fast the excess contrast at the edge of the sphere decays. β is set to 10% of the sphere radius. The volume fractions are defined by ϕ. The total volume of the colloid is defined as V_c which is $\frac{4}{3}\pi r^3$. The volume of the Gaussian coil is defined by the molecular weight ($M_w = 12500\ g * mol^{-1}$), Avogadro's number ($N_A = 6.02 \times 10^{23}\ molecules * mol^{-1}$) and the average density of protein ($\delta = 1.35 \times 10^{-24}\ g * \text{Å}^{-3}$). By varying the relative volume fractions of Gaussian coil to fuzzy colloid, we simulated the evolution of the form factor in response to a larger fraction of the measurement volume being occupied by assemblies / phase separated droplets (Supplementary Figure 6). The simulated form factor is a good qualitative representation of the data collected at later time points in laminar flow SAXS experiments at higher NaCl concentrations (Supplementary Figure 7, Figure 6).

Using the synthetic data, we tested the validity of the simplified assembly metric. It is clear theoretically that the zero-angle scattering is proportional to the volume fractions of both components. The intensity of the fuzzy colloid is also a function of the chosen distribution of radii. We assume that the experimental scattering is primarily determined by the volume fraction of fuzzy colloids based on two observations. First, while it is true that larger structures will have a higher scattering intensity, they also decay more rapidly with q. Therefore, we rule out contributions from droplets that are much larger than the maximum measurable distance as their intensity will have significantly decayed prior to the experimentally measurable angles. Second, the fusion and maturation of droplets continues in these systems on the time scale of tens of seconds to hours[9]. The presence of a plateau in the experimental data suggests that on that measured time scales the experiment is primarily sensitive to the fraction of frames that contain nanoscopic droplets. The model demonstrates that this condition holds true at the finite angles that are experimentally accessible if the assemblies are greater in size than the resolution of the experiment. Therefore, we use the intensity at small angles as a proxy for assembly (Supplementary Figure 6).

The mean assembly metric as a function of time was fit to a Weibull probability distribution which has previously been used to describe nucleation events[16]. The Weibull distribution is a phenomenological function that characterizes the probability of a transformation, in this case nucleation, as a function of time. The assembly metric data is fit to the cumulative probability distribution:

$$p(t) = A\left(1 - e^{-\left(\frac{t}{\lambda}\right)^{\kappa}}\right) \quad (8)$$

The distribution is characterized by two parameters. The shape parameter, λ, defines the width of the distribution or the total time for the system to completely transition. The stretching exponent, κ, defines the lag time. If $\kappa = 1$, the probability of nucleation will be constant and will only be a function of the density of protein yet to nucleate. Values of $\kappa > 1$ are indicative of a lag time and the probability of nucleation increases with time. The coefficient A was used to normalize the assembly metric. While some variation in intensity is expected between measurements, the theoretical maximum in the assembly metric is assumed to be of the same order between samples. Therefore only 80% variability is allowed in the A parameter between data sets. The parameters λ and κ obtained from fitting were used to plot the associated probability density function, or the derivative of the cumulative probability distribution:

$$P(t) = \frac{\kappa}{\lambda}\left(\frac{t}{\lambda}\right)^{\kappa-1} e^{-\left(\frac{t}{\lambda}\right)^{\kappa}} \quad (9)$$

All laminar flow data analysis and SAXS simulations were done using Matlab vR2019a.

Equilibrium dense phase SAXS measurements

Measurements of the dense phase of A1-LCD were performed at beamline 18ID-D (BioCAT) at the Advanced Photon Source at Argonne National Laboratory. The detector and hardware were identical to equilibrium SEC-SAXS measurements, with the exception that the sample chamber was replaced by a holder designed for sealed capillaries. Samples were prepared in open-ended 1 mm diameter quartz capillary tubes (Charles Supper Company). A phase separated sample was created in a 1.5 mL Eppendorf tube by adding 300 mM NaCl to 1 mL of 1 mM A1-LCD. The sample was centrifuged at 5000 x g for 5 minutes to collect the translucent dense phase in the bottom of the tube. The open end of the capillary was used to suck up the dense phase from the bottom of the Eppendorf tube using an attached Hamilton gas-tight syringe. The capillary was sealed on both ends using epoxy. The dense phase was manually centered in the beam and a series of 0.5 second exposures were taken. Due to the high concentration of the sample, the first exposure was sufficient for analysis and thus artifacts from radiation damage could be minimized. Buffer subtraction was performed by measuring only buffer in the same

capillary. Due to inherent mismatches due to manually centering the capillary in the beam, the buffer subtraction is imperfect and data at higher angles is unreliable. The data was analyzed using Supplementary Equation 3. The Gaussian chain R_G was similar to that from monomer equilibrium and time-resolved data (~26 Å). The correlation length related to the small-angle power-law scattering was >500 Å. Given the lack of curvature in the small angles, this value is only defined by a lower limit. The exponent relating to the power law decay was identical to time-resolved data (d=3.8) (Supplementary Figure 7).

Measurement of A1-LCD binodal

The phase boundaries for A1-LCD as a function of NaCl concentration were determined by UV absorption at 280 nm. A1-LCD stock solutions at 1.5 mM in 50 mM HEPES pH 7 buffer were passed through a 0.1 µm syringe filter and aliquoted into 20 µL aliquots if only light phase concentrations were to be acquired or 500 µL aliquots if dense phase concentrations were additionally acquired. The appropriate concentration of NaCl was then achieved by adding to each aliquot a defined volume of buffer containing 3 M NaCl in 50 mM HEPES pH 7. Additional 50 mM HEPES buffer with no NaCl was added to samples such that the total buffer addition (3 M NaCl buffer + 0 M NaCl buffer) was the same for each sample and thus the starting protein concentration was identical. After adjustment of NaCl concentrations, samples were allowed to equilibrate for 30 minutes followed by centrifugation for 10 minutes at 5000 x g. After centrifugation, samples were again allowed to equilibrate for 30 minutes at ambient room temperature (~22 °C).

Concentrations were determined by UV absorbance using a Nanodrop spectrophotometer (Thermo Fisher). Light phase measurements were done by mixing 5 µL of the supernatant with 5 µL of 6 M GdnHCl. Light phase measurements were performed in triplicate. Dense phase measurements were acquired by pipetting 1.5 µL of the pellet using a positive displacement pipette into 8.5 µL of 6 M GndHCl. Dense phase measurements were also performed in triplicate, but samples were removed from the same dense phase. Concentrations were determined from UV absorbance using the extinction coefficient 10430 $M^{-1}cm^{-1}$.

Fluorescence Correlation Spectroscopy fitting

Measurements in the dilute and dense phase were carried out using a conventional single-focus geometry in order to ensure focusing inside of the dense phase. For analysis of the corresponding correlations (Fig. 2), the following equations were fitted to each correlation curve:

$$G(t) = \frac{1}{N_{FCS}}\left(1+\frac{t}{\tau_D}\right)^{-1}\left(1+\frac{t}{s^2\,\tau_D}\right)^{-1/2} \qquad (10)$$

$$G(t) = \frac{1}{N_{FCS}\,(\sum_{i=1}^{n} f_i\,\varepsilon_i/\varepsilon_1)^2}\sum_{i=1}^{n} f_i\,(\varepsilon_i/\varepsilon_1)^2\,\left(1+\frac{t}{\tau_{Di}}\right)^{-1}\left(1+\frac{t}{s^2\,\tau_{Di}}\right)^{-1/2} \qquad (11)$$

where τ_D and τ_{Di} are is the characteristic diffusion time of the labeled molecule through the confocal volume, N_{FCS} is the apparent effective mean number of molecules in the confocal volume, and s represents the eccentricity of the confocal volume, f_i and ε_i are the fraction and molecular brightness of diffusing species i, with $\sum_{i=1}^{n} f_i = 1$, and n is the total number of diffusing species. The aspect ratio s parameter of the confocal volume was fixed to 6 based on independent determinations of the aspect ratio obtained from a 3D Gaussian fit to the emission profile of TetraSpeck^{TM} beads immobilized on a coverslip (Picoquant, Germany).

Fitting to correlation decays acquired inside the dense phase were adequately described by one species (Supplementary Equation 10a). In contrast, fitting to correlation decays in the dilute phase required the use of two diffusion species (Supplementary Equation 10b, with equal brightness). It is important to note that 2d-FCS measurements support that one single species is present in solution and that the two detected species result from imperfect fitting of the decay under the assumption of a Gaussian beam profile. An identical fit can be obtained by adding multiplicative terms that describe the departure from the Gaussian beam in terms of a triplet component, but this is found to alter the number of diffusive species in the region. Therefore, it is suboptimal if the concentration of the sample needs to be computed. Furthermore, the excitation power was chosen such that triplet contributions within the measured lag times were negligible, thus limiting the fitting parameters to the number of molecules *NFCS* and the diffusion time τ_D.

To obtain the dependence on NaCl concentration of the diffusion coefficient of Alexa488-labeled A1-LCD (Fig. S9), we used dual focus FCS. Analysis of the df-FCS traces (not shown) was performed according to Dertinger et al.[17] as implemented in the Fretica package developed by Ben Schuler and Daniel Nettels at the University of Zurich (https://schuler.bioc.uzh.ch/programs/). Interfoci distance was set to 0.40 μm adjusted to obtain D = 3.88 10-10 m2 s-1 for free Alexa 488 dye (value informed for diffusion at 25°C in Petrov & Schwille[18] is 4.14 10-10 m2 s-1 and was corrected to 22.5°C employing the Vogel

equation[19] with parameters for water, $\eta_{H_2O}\ (T)\ = 10^{-3} Exp\left[-3.7188\ -\ \frac{578.919}{T/K\ -\ 137.546}\right]$ in Pa.s units, with T given in Kelvins[9].

Numeric Simulations

We construct a nucleation model for droplets by extrapolating from a limiting case of the effective droplet model. In this effective droplet model we consider a single droplet of radius R with a homogeneous concentration that is equal to the equilibrium dense phase concentration, n^-. Outside the droplet we consider a spherically-symmetric steady-state diffusion concentration profile $n(r)$ where other droplets are very far away. This concentration approaches the current concentration of the bulk phase, n^∞, far away from the droplet. The concentration converges to that droplet's equilibrium saturation concentration, $\bar{n}^+$, immediately outside the droplet. Importantly, the droplet's equilibrium saturation concentration is dependent on the radius, R, of the droplet and can be estimated as $\bar{n}^+ \approx n^+(1 + 2\gamma/k_B T R n^-)$, where n^+ is the saturation concentration for an infinite sized droplet and γ is the surface tension[20]. The steady-state profile outside the droplet can thus be written as

$$n(r) = \frac{R}{r}(n^+(1 + \frac{2\gamma}{k_B T R n^-}) - n^\infty) + n^\infty\,, \tag{12}$$

and the flux of material into the droplet, $J = 4\pi R^2 D \nabla n(r = R)$ is given by

$$J = 4\pi R D(n^\infty - n^+(1 + \frac{2\gamma}{k_B T R n^-})), \tag{13}$$

where D is the is the diffusion coefficient outside the droplet. Rewriting the influx into the number of protein molecules in the droplet, N, gives us

$$\dot{N} = J = 4\pi D(\frac{3N}{4\pi n^-})^{1/3} n^\infty - 4\pi D((\frac{3N}{4\pi n^-})^{1/3} + \frac{2\gamma}{k_B T n^-})n^+\ . \tag{14}$$

The critical protein number, N_c, for the nucleation barrier is defined as the size of a droplet where $\dot{N} = 0$. This is given by

$$N_c = \frac{4\pi n^-}{3}(\frac{2\gamma n^+}{k_B T n^-(n^\infty - n^+)})^3\ . \tag{15}$$

All droplets larger than N_c will on average grow and all droplets smaller than N_c will on average shrink.

The droplet dynamics described in Supplementary Equation 14 can be thought of as two competing terms. The first term, proportional to the dilute concentration n^∞, is the growth rate for the droplet, and the second term, proportional to the interface concentration n^+ is the shrinkage rate for the droplet. Converting the continuous model of droplet size into a discrete model requires converting the fluxes into transition probabilities per time. These transition probabilities correspond to the probability that a droplet will grow or shrink by one protein molecule. For small integration time steps, Δt, the probability of growth and shrinkage are given by their flux times Δt,

$$\begin{aligned} P_{N\to N+1} &= 4\pi D(\frac{3N}{4\pi n^-})^{1/3} n^\infty \Delta t \ , \\ P_{N\to N-1} &= \begin{cases} 0, & \text{if } N = 1 \\ 4\pi D((\frac{3N}{4\pi n^-})^{1/3} + \frac{2\gamma}{k_B T n^-}) n^+ \Delta t, & \text{otherwise} \end{cases} . \end{aligned} \quad (16)$$

We can thus extend this to many coexisting clusters where the ith cluster has transition probability

$$\begin{aligned} P_{N_i\to N_i+1} &= 4\pi D(\frac{3N_i}{4\pi n^-})^{1/3} n^\infty \Delta t \ , \\ P_{N_i\to N_i-1} &= \begin{cases} 0, & \text{if } N_i = 1 \\ 4\pi D((\frac{3N_i}{4\pi n^-})^{1/3} + \frac{2\gamma}{k_B T n^-}) n^+ \Delta t, & \text{otherwise} \end{cases} , \end{aligned} \quad (17)$$

where N_i is the number of proteins in the ith cluster. Finally, we model the dilute phase concentration, n^∞, through the summation of all material that is in small clusters,

$$n^\infty = \frac{1}{V} \sum_i^M N_i \, \Theta(N_i - N_s) \ , \quad (18)$$

where V is the volume of the entire system, M is the total number of clusters, N_s is the threshold size for a small cluster being counted as part of the bulk solution, and Θ is the Heaviside function defined as

$$\Theta(N_i - N_s) = \begin{cases} 1, & N_i \le N_s \\ 0, & N_i > N_s \end{cases} . \quad (19)$$

We note that the growth or shrinkage of droplets will necessitate removing or adding clusters of size one from the system such that the $\sum N_i = N_{tot}$ where N_{tot} is the total number of proteins being simulated.

All numeric simulations were done using Matlab vR2020B.

Supplementary Note 3: Connecting nucleation rate to supersaturation

In classical nucleation theory one considers a change in free energy ΔG associated with forming a droplet of size R. ΔG consists of two terms, a volume term that describes the bulk phase separation behavior, and a surface term that describes the surface tension of a droplet (Equation 1, main text),

$$\Delta G = 4\pi R^2\gamma + \frac{4}{3}\pi R^3\epsilon \quad (20)$$

where R is the radius of the droplet, ϵ is the free energy difference per volume between inside and outside of the droplet, and γ is the surface tension of the droplet. The nucleation barrier R_C is defined as the size of the droplet with the highest free energy as this is the size that must be reached before growth is on average faster than shrinking,

$$R_C = \frac{2\gamma}{-\epsilon}\,. \quad (21)$$

We can compare this to the critical size calculated from the effective droplet model,

$$N_C = \frac{4}{3}\pi n^-\left(\frac{2\gamma n^+}{k_B T n^-(n^\infty - n^+)}\right)^3 , \quad (22)$$

where N_C is the critical number of proteins in the droplet for nucleation, n^- is the concentration inside the droplet, n^+ is the saturation concentration, and n^∞ is the concentration of the solution. Converting from the number of protein copies to the radius, $N = 4/3\pi R^3 n^-$, and equating the two models gives us

$$\epsilon = -\frac{k_B T n^-(n^\infty - n^+)}{n^+}\,. \quad (23)$$

Substituting the critical radius into our free energy gives us the height of the energy barrier to nucleate a droplet,

$$\Delta G_C = \frac{16}{3}\pi\gamma^3\left(\frac{n^+}{k_B T n^-(n^\infty - n^+)}\right)^2 . \quad (24)$$

Following classical nucleation, the nucleation rate Φ is proportional to the probability of a cluster being size N_C

$$\Phi \propto \exp\left(\frac{16}{3}\pi\left(\frac{\gamma}{k_B T}\right)^3\left(\frac{n^+}{n^-(n^\infty - n^+)}\right)^2\right) . \quad (25)$$

The degree of supersaturation is defined as $\sigma = n^{\infty} - n^{+}$ and therefore

$$\Phi \propto \exp(\frac{1}{\sigma^2}). \quad (26)$$

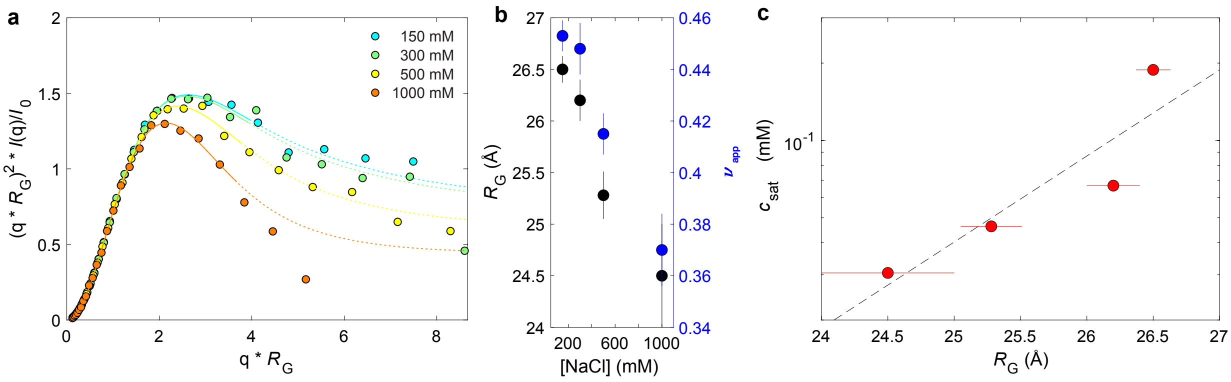

Supplementary Figure 1: A1-LCD is more compact at higher NaCl concentration. (a) Equilibrium SAXS data at NaCl concentrations ranging from 150 mM to 1000 mM. Data are displayed in dimensionless Kratky format and are logarithmically binned into 30 bins. The solid lines are fits to the IDR form factor[6] and the dashed lines are extrapolation from the IDR form factor model. Deviations in the extrapolation at high salt reflect both the difficulty of the form factor in fitting compact ensembles at larger values of q * R_G and the higher experimental uncertainty at high salt. **(b)** The R_G and apparent scaling exponent (ν_{app}) from the IDR form factor fit. Realistic uncertainty values for ν_{app} are likely higher than reported due to the poor fit of the model at larger values of q * R_G and high salt. Error bars are derived from the standard error of the fit to the molecular form factor. **(c)** The R_G values from the IDR form factor fit in A are correlated with c_{sat} shown in Figure 2b. Error bars are derived from the standard error of the fit to the molecular form factor. Data from panels a and b are replotted from Martin et al.[21].

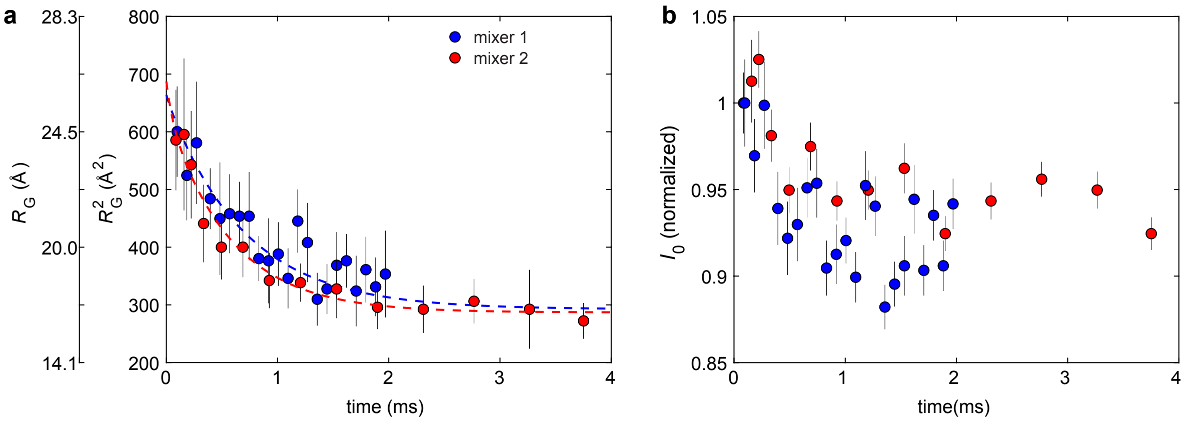

Supplementary Figure 2: Chaotic-flow cytochrome c refolding experiments. (a) The efficiency of mixing in chaotic-flow mixing experiments is controlled for by measuring the well-defined refolding rate for cytochrome c. Red and blue data points stem from two similar mixers used to collect data for A1-LCD and cytochrome c, respectively. The dashed lines are bi-exponential decays calculated using previously reported time constants (45 and 650 μs). Error bars represent the standard deviation of the fit plus the standard deviation of R_G from all compatible Guinier regions[22]. **(b)** The zero-angle scattering I_0 varies minimally during collapse. Error bars represent the standard deviation in the fit.

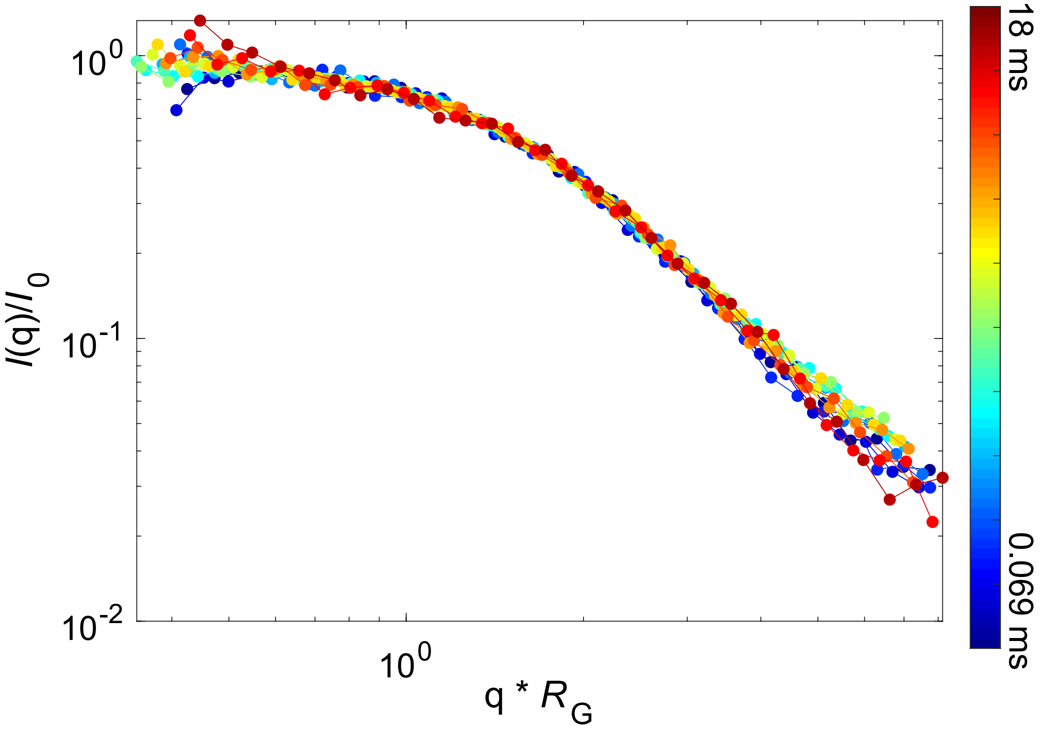

Supplementary Figure 3: Raw SAXS data from chaotic-flow mixing experiments. All data are normalized by I_0 and R_G calculated using the IDP form factor. This representation highlights the minimal evolution of the form factor over the course of the 18 ms experiment. The majority of changes are captured by the change in R_G.

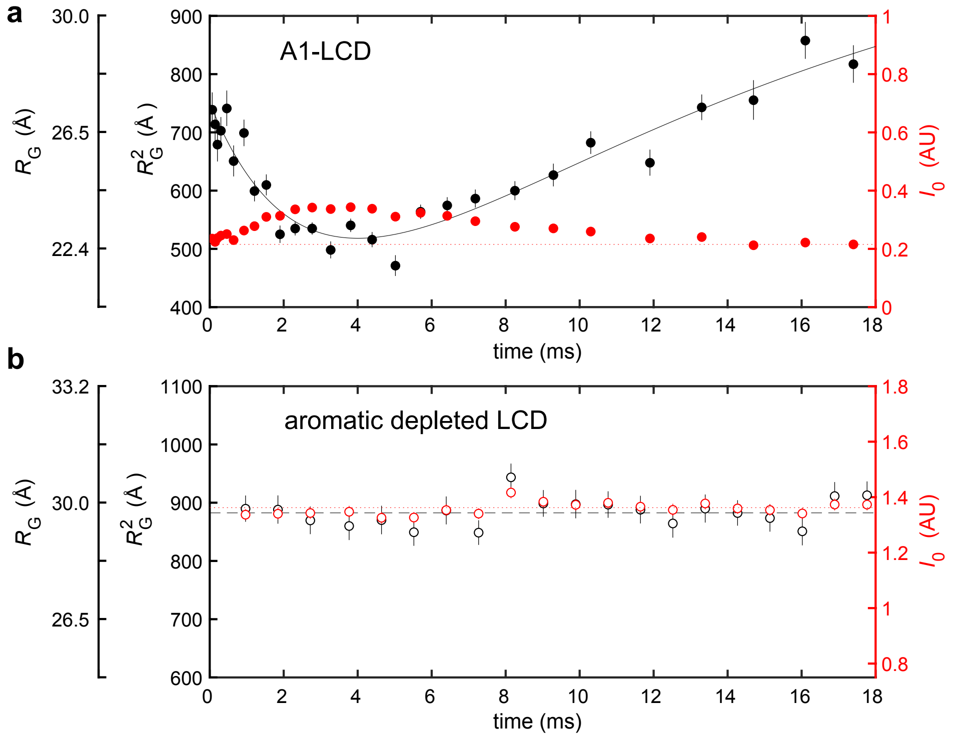

Supplementary Figure 4: A1-LCD chaotic-flow TR-SAXS experiments show anticorrelated R_G and I_0. (a) The black data points are replotted A1-LCD R_G values from Figure 3b. Overlaid are the measurements of I_0 in red extracted from the molecular form factor fit. The dashed red line is to guide the eye and is set to the final value of I_0. Error bars represent the standard error in the fit to the IDR form factor. **(b)** The open black data points are replotted aromatic-depleted LCD R_G values from Figure 3b along with I_0 values in red. Error bars represent the standard error in the fit to the IDR form factor. Measurements of A1-LCD and the aromatic depleted LCD were performed on different days with different x-ray flux. I_0 is not given in absolute units, and the values cannot be directly compared between samples.

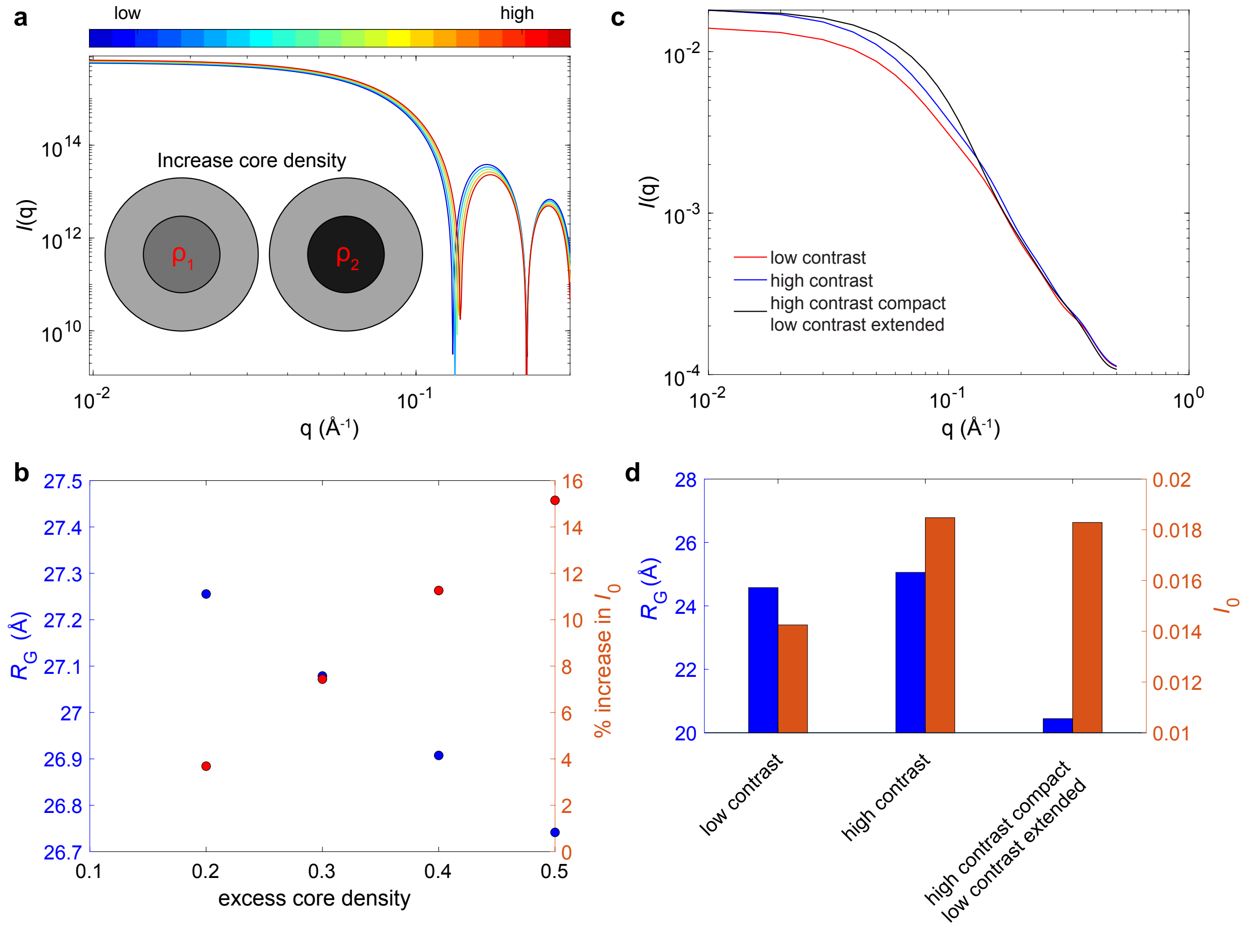

Supplementary Figure 5: Can solvation effects explain the increase in I_0 observed in TR-SAXS experiments of A1-LCD? (a) An increase in bound solvent can be qualitatively modeled by assuming a core-shell model in which the density of the core is increased to represent more solvent bound to the interior. **(b)** Increasing the core density results in an increase in I_0 and a decrease in R_G (calculated by Guinier fit to simulated data). **(c)** Solvation effects can additionally be modeled by changing the solvation shell thickness used in Crysol to calculate SAXS profiles on minimal ensembles of A1-LCD conformations. Shown are cases where "low" contrast is used for the whole ensemble, where "high" contrast is used for the whole ensemble, and where "high" contrast is used for compact conformations while "low" is used for extended conformations. "High" and "low" refer to values of 0.03 and 0.02 of the 'dro' parameter in Crysol, respectively. **(d)** Values of R_G and I_0 calculated from Guinier analysis of the simulated data in c.

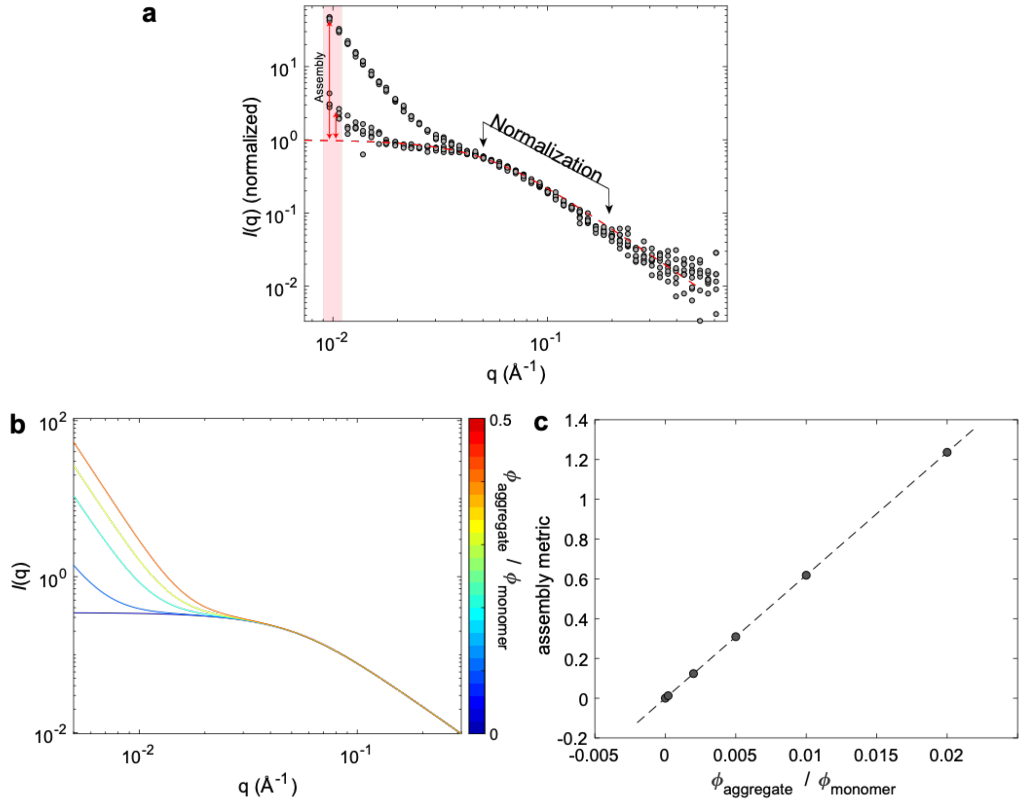

Supplementary Figure 6: The form factor of assembly data is modeled as a complex mixture of monomers and fuzzy colloids. (a) A schematic indicating how the assembly metric is calculated. Two sets of logarithmically smoothed raw data (from the 400 mM NaCl sample) representing time points from a minimally assembled and significantly assembled sample are shown as grey circles. The monomer form factor is shown as a red dashed line. Each set of raw data is normalized to the monomer form factor using the indicated region. The assembly is calculated as the average deviation from the monomer form factor calculated in the red shaded region. **(b)** The form factor is calculated by combining a Gaussian chain for the monomer with a polydisperse mixture of 'fuzzy colloids' representing phase separated assemblies. The polydispersity is generated by assuming a Gaussian distribution of radii centered on 50 nm. The relative volume fraction of monomer to assembly is then varied. **(c)** The assembly metric that is used to quantify experimental data was used to assess synthetic data. The assembly metric varies linearly with the relative volume fraction of assemblies, demonstrating that it is a useful metric.

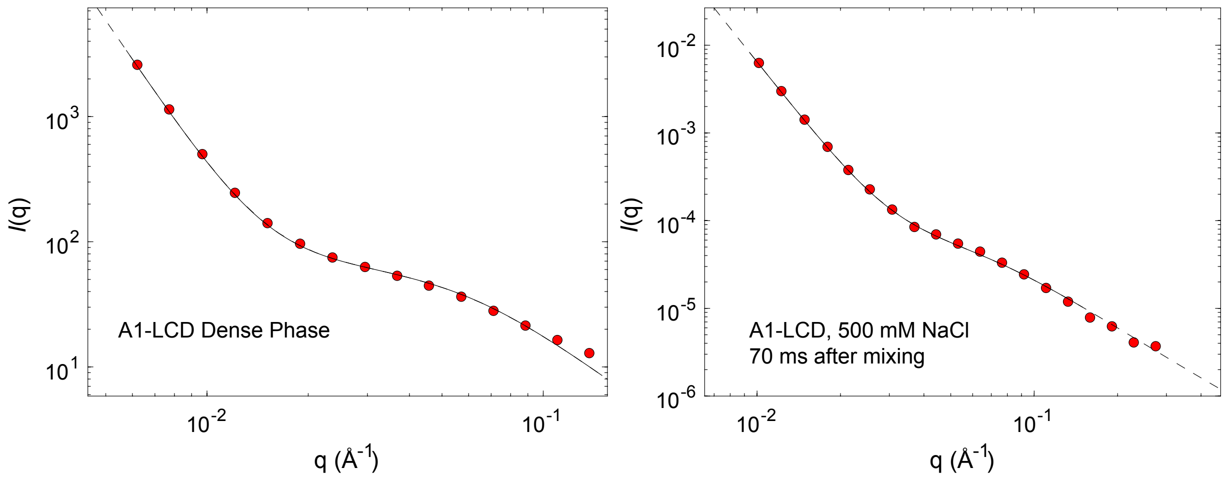

Supplementary Figure 7: The form factor of the dense phase of A1-LCD matches the form factor from late time points in laminar flow mixing experiments. The form factor of a sedimented dense phase (left) and late time points in the 500 mM NaCl laminar-flow experiment (right) are logarithmically smoothed into 20 bins and are shown as red circles. Data were fit independently to Supplementary Equation 3 (black line). The black line is solid in the region used in the fit and extrapolated regions are shown as a dashed line. The power law describing the scattering at small angles is d~3.8 and the lack of curvature in the small angles sets a minimum value for the correlation length of ~ 500 Å in both samples. The Gaussian chain region is characterized by an R_G of ~26 Å in both samples. The only difference in shape is due to the coefficients (A and B in Supplementary Equation 3) which are indicative of differences in the size of the interface contributing to the power law scattering. The dense phase sample is collected by centrifugation, and interfaces are thus expected to be small relative to a sample with suspended droplets.

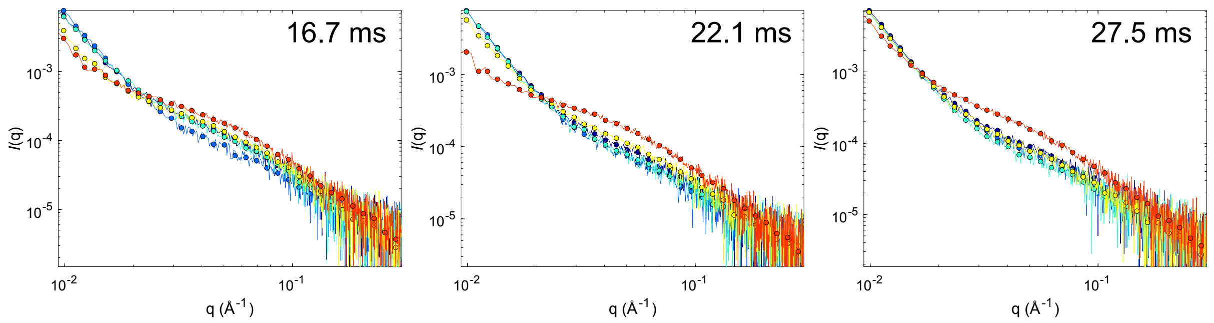

Supplementary Figure 8: Heterogeneity in the laminar flow TR-SAXS experiments is due to variability in the form factor and not experimental uncertainty. Unnormalized raw SAXS data from individual time points taken from the 500 mM NaCl experiment. In order to highlight the heterogeneity of the form factor, time points were selected around the maximum of the Weibull probability density distribution.

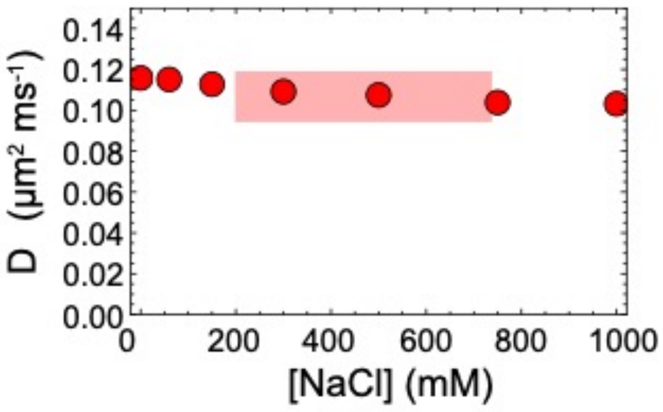

Supplementary Figure 9: Diffusion constants as a function of NaCl for dilute A1-LCD measured by 2f-FCS. Experiments were carried out on samples of 1 nM Alexa-488 labeled A1-LCD. The Diffusion constant shows a modest dependence on NaCl concentration between 0 and 1 M. All values between 0.2-0.8 M NaCl fall within the standard error for these three points. The region considered by TR-SAXS measurements and numerical simulations is designated with a red box.

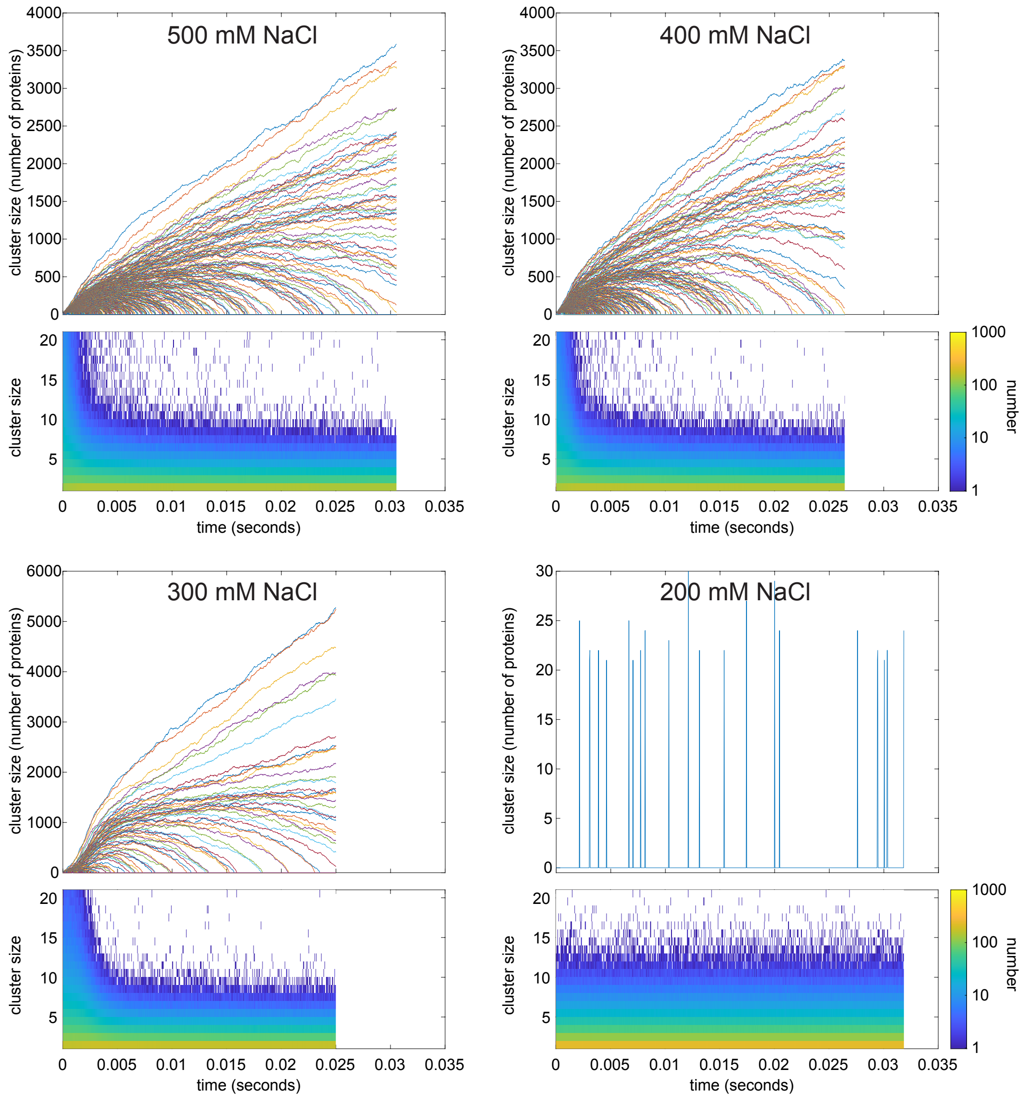

Supplementary Figure 10: Individual traces from simulations of nucleation. Sample data from simulations covering the same NaCl concentrations used in experiments (500, 400, 300 and 200 mM). For each NaCl concentration the top figure shows the evolution of individual clusters growing or shrinking after reaching a threshold size of 20 molecules. The lower figure shows the evolution of the cluster size distribution below the 20-molecule threshold. In all cases where mesoscopic assemblies form, the small cluster distributions converge. At 200 mM NaCl, in the absence of nucleation, the distribution skews to larger sized (< 20) clusters due to the higher concentration in the homogeneous phase.

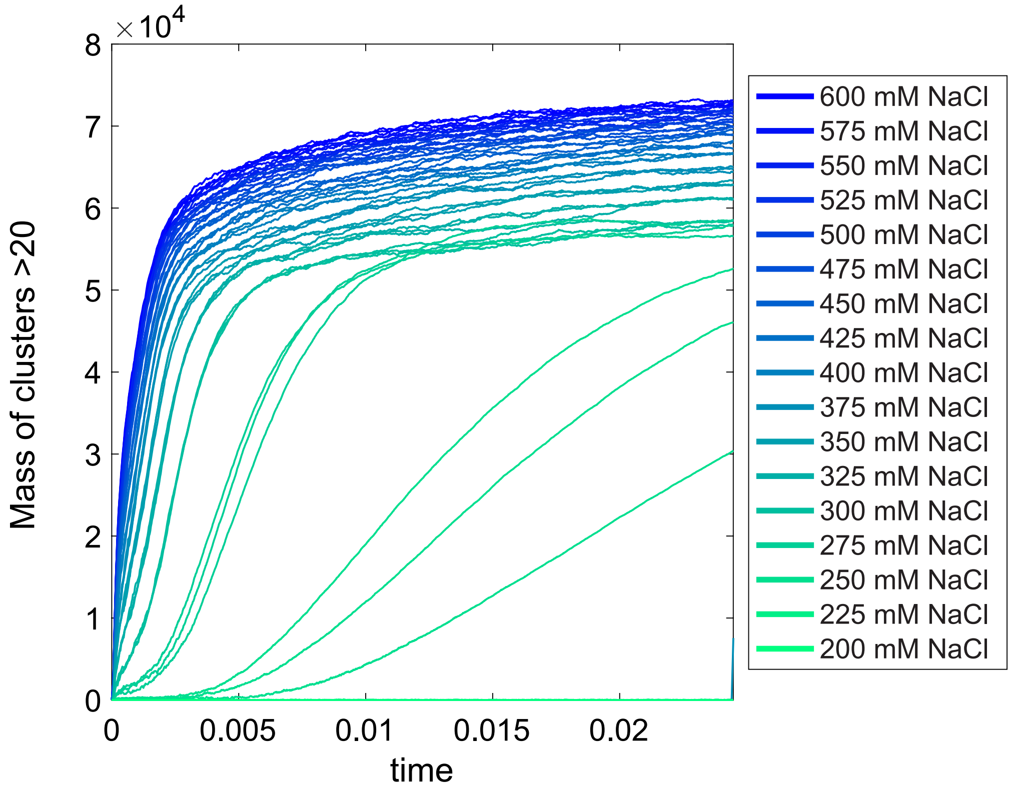

Supplementary Figure 11: The time evolution of the mass of clusters in simulations. The total mass in clusters of more than 20 molecules as a function of time. Figure 8b in the main text shows the average of three simulations while all three simulations for each NaCl concentration are shown here.

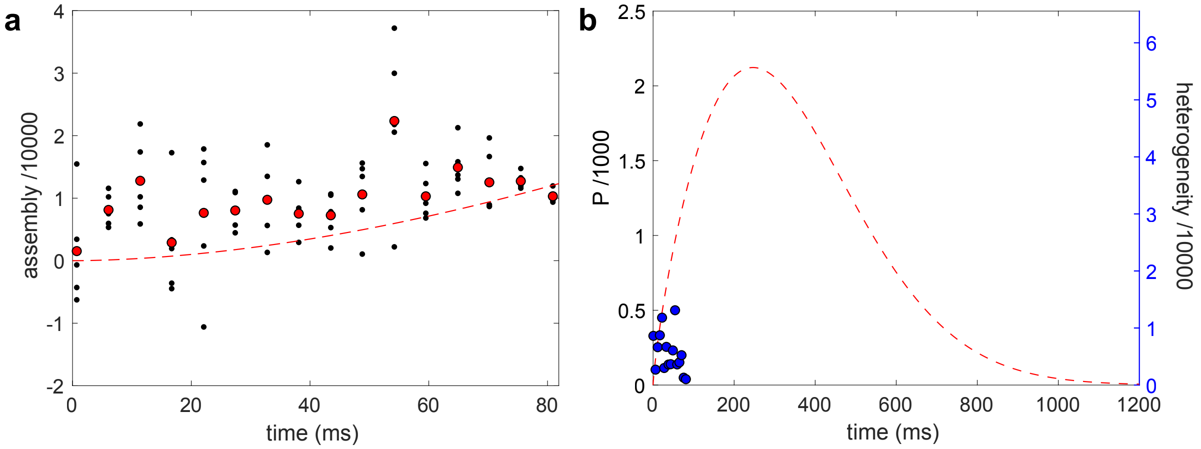

Supplementary Figure 12: Nucleation slows near the binodal boundary. (a) Assembly data for 200 mM NaCl. Black dots are individual measurements and red circles are the mean values. The red dashed line is the cumulative probability distribution calculated from the extrapolated parameters in A. **(b)** The probability density distribution calculated from the extrapolated parameters in A. The blue circles represent the experimentally determined heterogeneity.

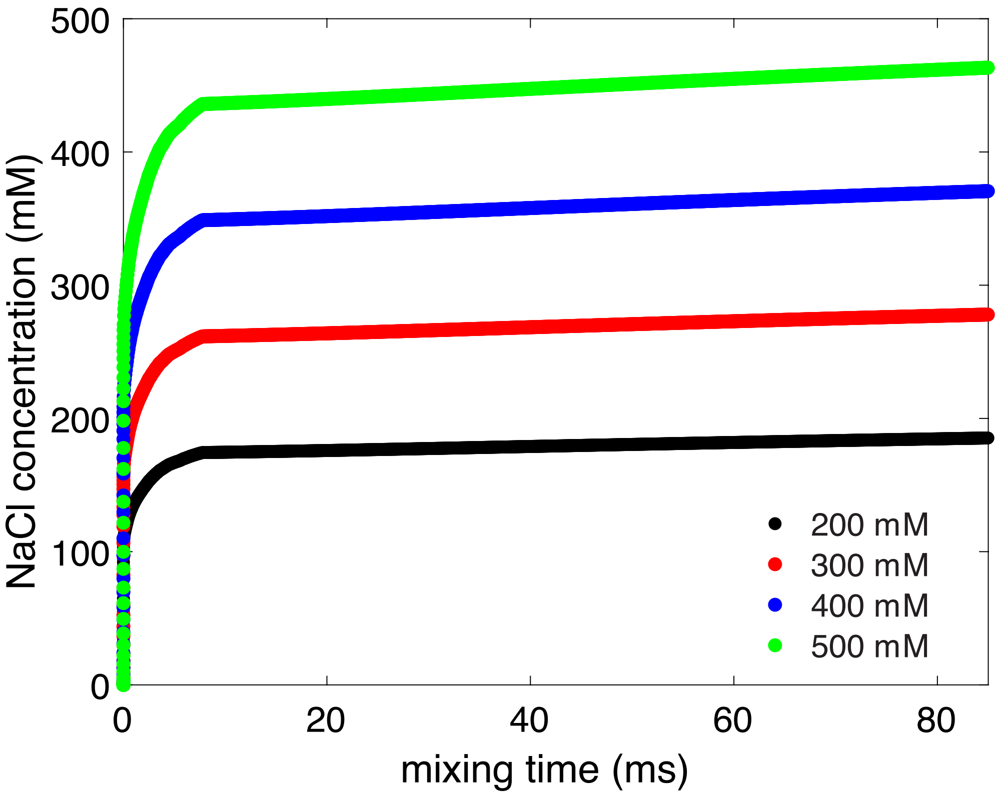

Supplementary Figure 13: Calculated increase of NaCl concentration as a function of mixing time in laminar flow experiments. NaCl concentration over the course of the experiment can be calculated based on diffusion constants and the mixer geometry. Mixing is incomplete on the timescale of the experiment, and the actual concentration is shown. After ~5 ms the majority of mixing is complete, and the concentration varies by only 5%.

Supplementary Table 1: DNA sequences of A1-LCD and the aromatic-depleted LCD.

A1-LCD	1	ATGTCGTACT ACCATCACCA TCACCATCAC CTCGAATCAA CAAGTTTGTA CAAAAAAGCA
	61	GGCTTCGAAA ACCTGTATTT TCAGGGCAGC ATGGCTAGTG CTTCATCCAG CCAAAGAGGT
	121	CGAAGTGGTT CTGGAAACTT TGGTGGTGGT CGTGGAGGTG GTTTCGGTGG GAATGACAAC
	181	TTCGGTCGTG GAGGAAACTT CAGTGGTCGT GGTGGCTTTG GTGGCAGCCG TGGTGGTGGT
	241	GGATATGGTG GCAGTGGGGA TGGCTATAAT GGATTTGGTA ATGATGGAAG CAATTTTGGA
	301	GGTGGTGGAA ATTACAACAA TCAGTCTTCA AATTTTGGAC CCATGAAGGG AGGAAATTTT
	361	GGAGGCAGAA GCTCTGGCCC CTATGGCGGT GGAGGCCAAT ACTTTGCAAA ACCACGAAAC
	421	CAAGGTGGCT ATGGCGGTTC CAGCAGCAGC AGTAGCTATG GCAGTGGCAG AAGATTTTAA
Aromatic-depleted LCD	1	ATGTCGTACT ACCATCACCA TCACCATCAC CTCGAATCAA CAAGTTTGTA CAAAAAAGCA
	61	GGCTTCGAAA ACCTGTATTT TCAGGGCAGC ATGGCCAGCG CCAGCAGCAG CCAGAGAGGC
	121	AGAAGCGGCT CTGGCAATAG CGGCGGAGGA AGAGGCGGCG GATTCGGCGG CAATGACAAT
	181	TCTGGCAGAG GCGGCAACAG CAGCGGCAGA GGGGGATTTG GAGGCTCTAG AGGCGGAGGC
	241	GGAAGTGGCG GAAGCGGCGA CGGCTATAAT GGCAGCGGCA ACGACGGCAG CAATTCCGGG
	301	GGAGGCGGCA GCTCCAACGA CTTCGGCAAC TCCAACAACC AGAGCAGCAA CAGCGGCCCC
	361	ATGAAGGGCG GCAACTTTGG CGGCAGATCT AGCGGAGGAT CTGGCGGAGG GGGCCAGTAC
	421	TCTGCCAAGC CCAGAAATCA GGGCGGCAGC GGCGGATCTT CCAGCAGCTC TAGCTCTGGC
	481	TCCGGCAGGC GGAGCTAA

Supplementary References

1 Fischetti, R. *et al.* The BioCAT undulator beamline 18ID: a facility for biological non-crystalline diffraction and X-ray absorption spectroscopy at the Advanced Photon Source. *J Synchrotron Radiat* **11**, 399-405, doi:10.1107/S0909049504016760 (2004).

2 Hopkins, J. B., Gillilan, R. E. & Skou, S. BioXTAS RAW: improvements to a free open-source program for small-angle X-ray scattering data reduction and analysis. *J Appl Crystallogr* **50**, 1545-1553, doi:10.1107/S1600576717011438 (2017).

3 Peran, I. *et al.* Unfolded states under folding conditions accommodate sequence-specific conformational preferences with random coil-like dimensions. *Proc Natl Acad Sci U S A* **116**, 12301-12310, doi:10.1073/pnas.1818206116 (2019).

4 Halloran, K. T. *et al.* Frustration and folding of a TIM barrel protein. *Proc Natl Acad Sci U S A* **116**, 16378-16383, doi:10.1073/pnas.1900880116 (2019).

5 Petoukhov, M. V., Konarev, P. V., Kikhney, A. G. & Svergun, D. I. ATSAS 2.1 -- towards automated and web-supported small-angle scattering data analysis. *Journal of Applied Crystallography* **40**, s223--s228, doi:10.1107/S0021889807002853 (2007).

6 Riback, J. A. *et al.* Innovative scattering analysis shows that hydrophobic disordered proteins are expanded in water. *Science* **358**, 238-241, doi:10.1126/science.aan5774 (2017).

7 Mertens, H. D. & Svergun, D. I. Structural characterization of proteins and complexes using small-angle X-ray solution scattering. *J Struct Biol* **172**, 128-141, doi:10.1016/j.jsb.2010.06.012 (2010).

8 Kathuria, S. V. *et al.* Microsecond barrier-limited chain collapse observed by time-resolved FRET and SAXS. *J Mol Biol* **426**, 1980-1994, doi:10.1016/j.jmb.2014.02.020 (2014).

9 Martin, E. W. *et al.* Valence and patterning of aromatic residues determine the phase behavior of prion-like domains. *Science* **367**, 694-699, doi:10.1126/science.aaw8653 (2020).

10 Guinier, A. & Fournet, G. r. *Small-angle scattering of X-rays*. (Wiley, 1955).

11 Svergun, D., Barberato, C. & Koch, M. H. J. CRYSOL -- a Program to Evaluate X-ray Solution Scattering of Biological Macromolecules from Atomic Coordinates. *Journal of Applied Crystallography* **28**, 768--773, doi:10.1107/S0021889895007047 (1995).

12 Kalkowski, J. *et al.* In Situ Measurements of Polymer Micellization Kinetics with Millisecond Temporal Resolution. *Macromolecules* **52**, 3151-3157, doi:10.1021/acs.macromol.8b02257 (2019).

13 Hammouda, B., Ho, D. L. & Kline, S. Insight into Clustering in Poly(ethylene oxide) Solutions. *Macromolecules* **37**, 6932-6937, doi:10.1021/ma049623d (2004).

14 Debye, P. Molecular-weight Determination by Light Scattering. *The Journal of Physical and Colloid Chemistry* **51**, 18-32, doi:10.1021/j150451a002 (1947).

15 Stieger, M., Pedersen, J. S., Lindner, P. & Richtering, W. Are Thermoresponsive Microgels Model Systems for Concentrated Colloidal Suspensions? A Rheology and Small-Angle Neutron Scattering Study. *Langmuir* **20**, 7283-7292, doi:10.1021/la049518x (2004).

16 Sear, R. P. Quantitative studies of crystal nucleation at constant supersaturation: experimental data and models. *CrystEngComm* **16**, 6506-6522, doi:10.1039/C4CE00344F (2014).

17 Dertinger, T. *et al.* Two-focus fluorescence correlation spectroscopy: a new tool for accurate and absolute diffusion measurements. *Chemphyschem* **8**, 433-443, doi:10.1002/cphc.200600638 (2007).

18 Petrov, E. P. & Schwille, P. in *Standardization and Quality Assurance in Fluorescence Measurements II: Bioanalytical and Biomedical Applications* (ed Ute Resch-Genger) 145-197 (Springer Berlin Heidelberg, 2008).

19 Rubinstein, M. & Colby, R. H. *Polymer Physics*. (Oxford University Press, 2003).

20 Weber, C. A., Zwicker, D., Julicher, F. & Lee, C. F. Physics of active emulsions. *Rep Prog Phys* **82**, 064601, doi:10.1088/1361-6633/ab052b (2019).

21 Martin, E. W. *et al.* Interplay of folded domains and the disordered low-complexity domain in mediating hnRNPA1 phase separation. *Nucleic Acids Res*, doi:10.1093/nar/gkab063 (2021).

22 Petoukhov, M. V., Konarev, P. V., Kikhney, A. G. & Svergun, D. I. ATSAS 2.1 - towards automated and web-supported small-angle scattering data analysis. *Journal of Applied Crystallography* **40**, s223-s228, doi:doi:10.1107/S0021889807002853 (2007).
